# CD2 Promotes Human Natural Killer Cell Membrane Nanotube Formation

**DOI:** 10.1371/journal.pone.0047664

**Published:** 2012-10-24

**Authors:** Colin J. Comerci, Emily M. Mace, Pinaki P. Banerjee, Jordan S. Orange

**Affiliations:** 1 Department of Pediatrics, Children’s Hospital of Philadelphia Research Institute, Philadelphia, Pennsylvania, United States of America; 2 Department of Pediatrics, Center for Human Immunobiology, Baylor College of Medicine, Houston, Texas, United States of America; Hannover Medical University (MHH), Germany

## Abstract

Membrane nanotubes are thin membranous projections that physically connect two cells. While nanotubes have been studied in human natural killer (NK) cells and are implicated in aiding NK cell cytotoxic function, requirements for their formation to susceptible target cells remain incompletely understood. Here we demonstrate that the CD2-CD58/48 receptor-ligand interaction promotes and is required for nanotube formation in human NK cells. In the CD2^−^ NK cell line YTS, a stable CD2 expression variant enabled effective nanotube formation, and was associated with better cytotoxic function. Importantly, only interactions between an NK cell and a susceptible target cell were associated with multiple nanotubes and the number of nanotubes was inversely correlated with their length. Quantitative live cell fluorescence microscopy of CD2 nanotubes revealed time-dependent enrichment and localization of CD2 to the nanotube tip, and blocking CD2 receptor-ligand interactions prevented nanotube formation. Increased nanotube formation was not simply a feature of receptor-ligand pairing, as a KIR-MHC interaction in the same cell line system failed to promote nanotube formation. Additionally, blocking LFA-1-ICAM and 2B4-CD48 receptor-ligand interactions failed to inhibit nanotube formation. Thus only specific receptor-ligand pairs promote nanotubes. CD2 also promoted nanotube formation in *ex vivo* NK cells suggesting that CD2 plays a crucial role in the generation of nanotubes between an NK cell and its target.

## Introduction

Natural Killer (NK) cells are lymphocytes of the innate immune system that recognize and kill virally infected and tumorigenic cells. While NK cells can indirectly promote immune function via cytokine release and costimulation of other immune cells, direct NK cell cytotoxicity requires stable contact with target cells; in other words, transient interaction between an NK cell and its target must be stabilized by cell-cell adhesion forces prior to cytolysis [1]. One such adhesion force is provided via receptor-ligand interactions, some of which can also promote activation signaling like that of the NK cell receptors CD2 and its cognate ligands CD58 and CD48 [2], or LFA-1 and its cognate ligands ICAM-1 through 5 [3], [4]. Upon appropriate signals of stress and other activating signals from the target cell, a favorable balance of receptor-ligand interactions promotes formation of the NK cell immunological synapse (NKIS) which can further support activation signaling and cellular reorganization [5]. Downstream signaling directs the polarization of the microtubule organizing center and associated lytic granules to the immunological synapse providing a means for directed secretion and lysis of the target cell of interest [6].

Given NK cells’ lethal potential, it is paramount that they can effectively target infected and transformed cells, while sparing healthy cells. This specificity is provided via particular receptor-ligand interactions. Some receptor-ligand interactions, such as CD2-CD58/48 [7], activate NK cells and promote signaling that drives directed secretion and cytolysis [8]. Other receptors, such as killer-cell immunoglobulin-like receptor 2DL1 (KIR2DL1) [9], recognize determinants of self (e.g. HLA-Cw4) and inhibit NK cytotoxicity. Both activating and inhibiting receptor-ligand interactions require contact between NK and target cells, and both, in turn, provide some degree of adhesive force. These adhesion forces, in combination with appropriate activating or inhibitory signals, help to regulate NK cell cytotoxic function [6].

Regulation of NK cell cytotoxicity, however, involves more than just simple receptor-ligand interactions. As an added complexity, numerous thin membranous tethers, known as membrane nanotubes, form between NK cells and their targets. Originally described to enable intercellular traffic [10], nanotubes (NT) range in size from 20–200 nm in diameter and extend up to several cell diameters in length [10], [11]. They can connect a variety of immune cells, including T cells, T cells and their target cells, dendritic cells, NK cells, and NK cells and their target cells [11]. NTs are believed to perform a variety of functions, including assisting in intercellular communication by transporting vesicles and membrane proteins [11], [12], [13], transmitting calcium fluxes [14], and even transporting viruses between cells [12], [15].

Primary human NK cells and the human NK cell tumor line NKL readily form NTs with themselves and various target cells [16]. Functionally, NK cell NTs facilitate cytotoxic function both by aiding in the retrieval of motile target cells to form an NK cell immunological synapse (approximately 12% of NK cell cytotoxic events) and by transporting lytic granules along their length to mediate long-distance killing (approximately 5.4% of NK cell cytotoxic events) [16]. The specifics of NT formation, however, remain incompletely understood. Here we identify CD2 as a crucial promoter of NK cell NT formation.

## Materials and Methods

### Ethics Statement

All human samples were obtained using written informed donor consent and were used with the approval of the Children’s Hospital of Philadelphia and/or Baylor College of Medicine Institutional Review Board for the Protection of Human Subjects.

### Cell lines and ex vivo NK Cells

Immortalized NK cell lines derivative from the parental YTS cell line stably expressing a myosin IIA-GFP fusion protein (YTS-MyoIIA-GFP) [17], a CD2-GFP fusion protein (YTS-CD2-GFP) [18], or a KIR2DL1-GFP fusion protein (YTS-KIR-GFP) [9] were used as model NK cell systems. 721.221 B-lymphoblastoid cells (721), and 721.221 cells stably expressing HLA-Cw3 (721-Cw3) or HLA-Cw4 (721-Cw4) [19] were used as target cells (TC).

Ex vivo NK (eNK) cells were prepared either from whole blood obtained from volunteer donors by negative depletion using the RosetteSep human NK cell isolation reagent (StemCell Technologies), or from anonymized leukophoresis-derived PBMC via negative depletion using magnetic particles and the NK Cell Isolation Kit (Miltenyi Biotech). Alternatively, PBMCs were prepared from whole blood using centrifugation through Ficoll-Paque Plus lymphocyte isolation medium (Amersham Bioscience). eNK cells in PBMCs were identified as CD56^+^CD3^−^ and then sorted into CD2^+^ and CD2^−^ populations using a non-blocking CD2 antibody (BD Bioscience, clone L303.1) [20] and a Coulter MoFlo (Beckman Coulter). In all cases, cells were used immediately following preparation.

### Plate Coating

Anti-CD48 (BD Biosciences) or anti-CD20 (Rituximab, Genentech) were diluted to 8 µg/mL in PBS or in 0.2 µm filter-sterilized 10 µg/mL human fibronectin (Collaborative Biomedical Products or BD) in PBS prior to addition to ΔT dishes (Bioptechs). Only certain experiments were performed with added human fibronectin (FN) as specified. Dishes were incubated at 37°C for one hour in anti-CD48/PBS solution, or left overnight at 4°C in anti-CD48 or anti-CD20/fibronectin solution and then washed 3 times with PBS. Prior to use in experiments, fibronectin coated dishes were blocked with 0.2 µm filter-sterilized 3% BSA in PBS for one hour at room temperature.

### IL-2 Activation

YTS-MyoIIA-GFP cells were incubated for 24 hours in RPMI 1640 with 10% FCS supplemented with 125 U/mL human recombinant IL-2 (NIH AIDS reagents program).

### Live Cell Microscopy

NK cells (YTS-MyoIIA-GFP, YTS-CD2-GFP, YTS-KIR-GFP) and target cells (721, 721-Cw3, 721-Cw4) were washed and resuspended in RPMI 1640 with 10% FCS. eNK cells were labeled using the PKH26 red fluorescent dye (Sigma-Aldrich), and resuspended in RPMI 1640 with 10% FCS. For time course imaging, target cells were adhered to a coated ΔT dish for 20 minutes at 37°C. NK cells were added to the ΔT dish at a NK:TC ratio of 1.5∶1. Cells were imaged in a single z-plane using an Olympus IX-81 spinning disk confocal microscope, with Hamamatsu EM-CCD camera and Volocity Software (Perkin Elmer). Images were captured at maximum speed for the required exposure times (∼3 images/min) with both differential interference contrast microscopy and 488 nm or 568 nm excitation while the dishes were maintained at 37°C using a ΔT dish heater unit (Bioptechs). For single time point analyses, NK cells and target cells were incubated in a coated ΔT dish for 30 minutes at 37°C. After scanning planes for NT presence, cells were imaged in a single z-plane as above, or using a Zeiss Axio Observer Z1 with Yokogawa CSU10 spinning disk, Hamamatsu Orca-AG camera and Volocity Software for LFA-1 and 2B4 blocking antibody experiments. Images obtained using the Zeiss microscope were obtained using transmitted light and 488 nm excitation with cells maintained in a ΔT dish heater unit. 3D images were obtained using the Zeiss microscope at a *z*-axis spacing of 0.2 µm. For blocking antibody experiments, YTS-CD2-GFP cells were incubated with 10 µg/mL of biotinylated mouse IgG1 anti-human CD2 (BioLegend, clone RPA-2.10) [20], [21], biotinylated mouse IgG1 isotype control (BD), purified mouse IgG1 anti-human 2B4 (BioLegend, clone C1.7) [21], mouse IgG1 anti-human LFA-1 (clone TS1/22) [22], [23], or mouse IgG1 isotype control (BioLegend) for 15 minutes at 37°C prior to incubation on ΔT dishes.

### ^51^Cr Release Cytotoxicity Assay

Parental YTS or YTS-CD2-GFP cells were synchronized for 3 weeks by culturing in RPMI 1640 with 10% FCS. NK cell cytotoxic function was measured using a 4 hour ^51^Cr release assay as described previously [24].

### Data Analysis

**Figure 1:**
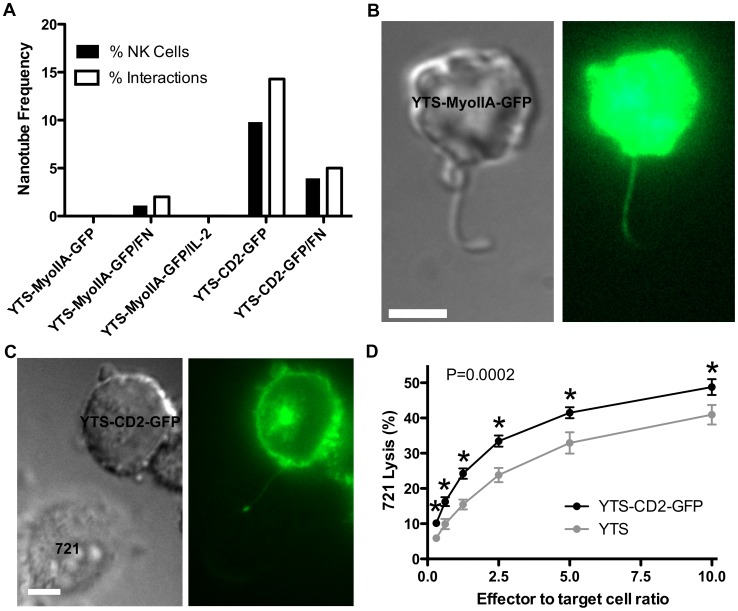
YTS-CD2-GFP but not YTS-MyoIIA-GFP NK cells form stable NTs. (A) The frequency of NT formation between NK cells and 721 cells was calculated as a percentage of NK cells forming NTs (black bars) and as a percentage of NK and 721 cell interactions leading to NT formation (white bars). See Materials and Methods for a detailed description of frequency calculations. YTS-MyoIIA-GFP cells were imaged with 721 cells on anti-CD48 coated plates, anti-CD48 and fibronectin (FN) coated plates, and on anti-CD48/FN coated plates with IL-2 pre-activation (n>100); YTS-CD2-GFP cells were imaged with 721 cells on anti-CD48 coated plates and on anti-CD48/FN coated plates (n>50). (B) YTS-MyoIIA-GFP NTs broke quickly, leaving thick projections ∼400 nm in diameter. (C) YTS-CD2-GFP NTs had a diameter of ∼100 nm and stably connected NK and target cells, sometimes for longer than 2 hours. (D) Cytotoxicity against 721 target cells is enhanced in YTS-CD2-GFP (black) cells in comparison to YTS (gray) cells (n = 3). (Scale bars: 5 µm.)

Image analysis was conducted using Volocity software (Perkin Elmer). For Fig. 1A, frequency of NK to target cell NTs was calculated as a percentage of NK cells (NK cells with NTs divided by the total number of NK cells observed), or as a percentage of cell interactions (where an interaction is defined as a NK to target cell contact lasting longer than a minute). For other figures, NT frequency was calculated by dividing the number of NK cells containing NTs, either NK to target cell NTs (NK-TC NT) or NK to NK cell NTs (NK-NK NT), by the total number of NK cells observed in single time point images. For the kinetic assessment of NTs, photobleaching was corrected using an exponential curve fit to the total cell fluorescent intensity (cell body and NT). Videos were then normalized to a relative time scale, ranging from 0 to 1. Time was split into 21 time bins (0.05 units), and the average value for the fluorescence or area of each NT tip was calculated for each time bin. Graphs represent the average of these values for all NTs. Trend lines were created using a linear regression in Prism (GraphPad). Time weighted averages were determined by calculating the fraction of time that a NT lasts compared to all of the NTs (the sum of all NT times), multiplying this fraction by the fluorescent value (or other measurement) of interest, and then summing across all NTs.

### Statistical Evaluations

**Figure 2:**
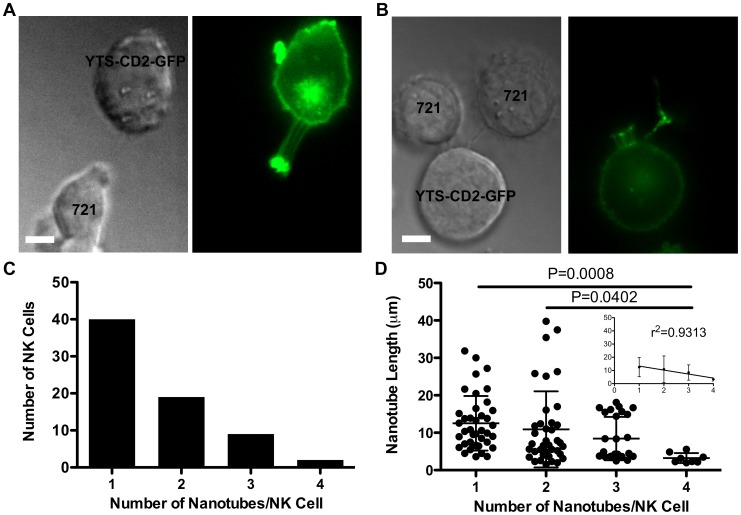
YTS-CD2-GFP cells can simultaneously form multiple NTs. YTS-CD2-GFP cells were incubated with 721 cells for 30 minutes on anti-CD48 coated plates then imaged live using a spinning disc confocal microscope at a single time point. (A) Multiple NTs form between a single NK and target cell, or (B) connect an NK cell to multiple target cells. (C) The number of NK cells forming multiple NTs is inversely proportional to the number of NTs per cell (n>100 NTs). (D) As the number of NTs per cell increases, the average NT length decreases (1–3 NTs/Cell: n>25 NTs; 4 NTs/Cell: n>5 NTs). (Scale bars: 5 µm.)

**Figure 3:**
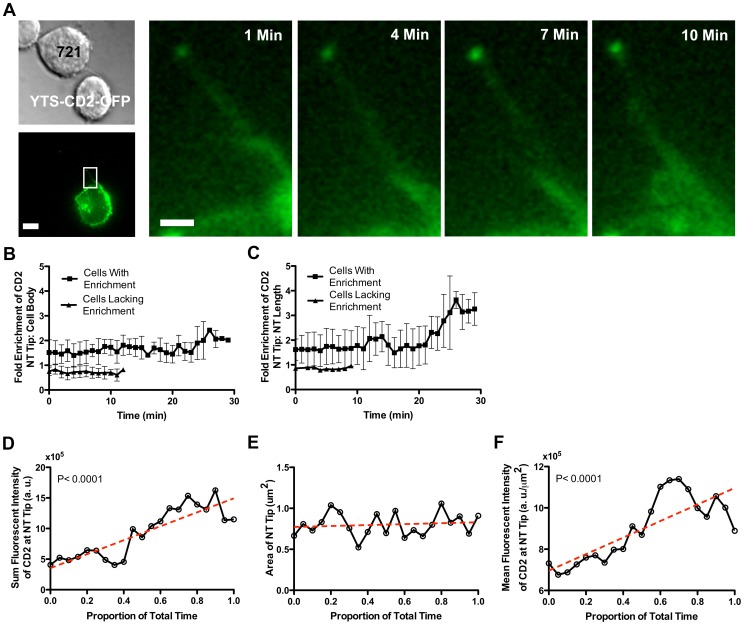
CD2 localizes to the distal tip of the NK cell NT. (A) A bright patch of CD2 fluorescence is seen at the distal tip of a YTS-CD2-GFP cell NT. Magnification of the boxed region depicts the NT tip becoming brighter over time as CD2 accumulates. (B) 65% of NK cell NTs exhibited an enrichment of CD2 at the NT tip as compared to the cell body. Enrichment persisted for the life of the NT (n>100 NTs). (C) Similarly, 85% of NK cell NTs exhibit enrichment of CD2 at the tip as compared to the overall length of the NT (n>100 NTs). (D), (E) and (F) Videos of NTs that show CD2 enrichment of the tip compared to the length of the NT were photo bleach-corrected, normalized to a unit time scale, time-binned, and averaged. Both sum (D) and mean fluorescent intensity (F) increased significantly over time, indicating that CD2 accumulates at the junction over time. (E) Tip area increased, but only marginally over time. Trend lines are shown as red dashed lines (n = 13 NTs). (Scale bars: 5 µm, Inset: 1 µm.)

**Figure 4:**
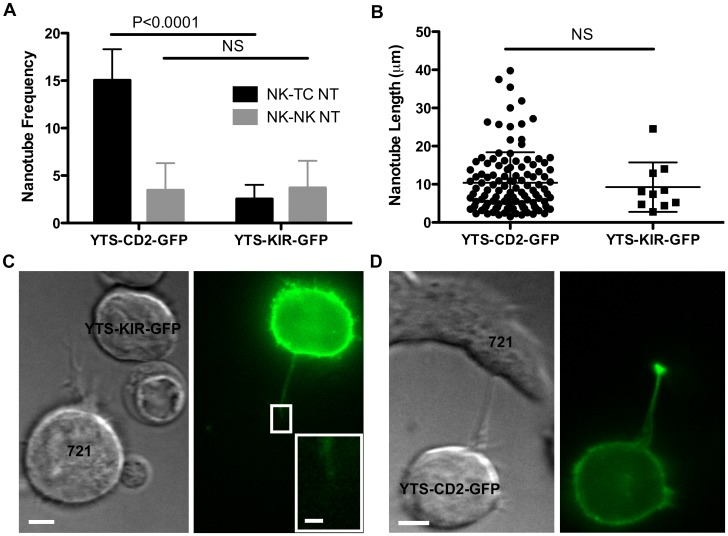
YTS-CD2-GFP cells form more NTs than YTS-KIR-GFP cells. (A) YTS-CD2-GFP and YTS-KIR-GFP cells were incubated with 721 cells for 30 minutes on anti-CD48 coated plates, then single images were obtained using live-cell confocal microscopy and scanning of z-planes to ensure NT detection. Bars show the percentage of NK cells exhibiting NK to target cell NTs (black) and NK to NK cell NTs (grey). See Materials and Methods section for a detailed description of frequency calculations. (B) Average NT length did not vary. Representative images of (C) YTS-KIR2DL1-GFP, with inset showing a lack of KIR enrichment at the tip and (D) YTS-CD2-GFP NTs. (n>320 cells) (Scale bars: 5 µm, Inset: 1 µm.)

**Figure 5:**
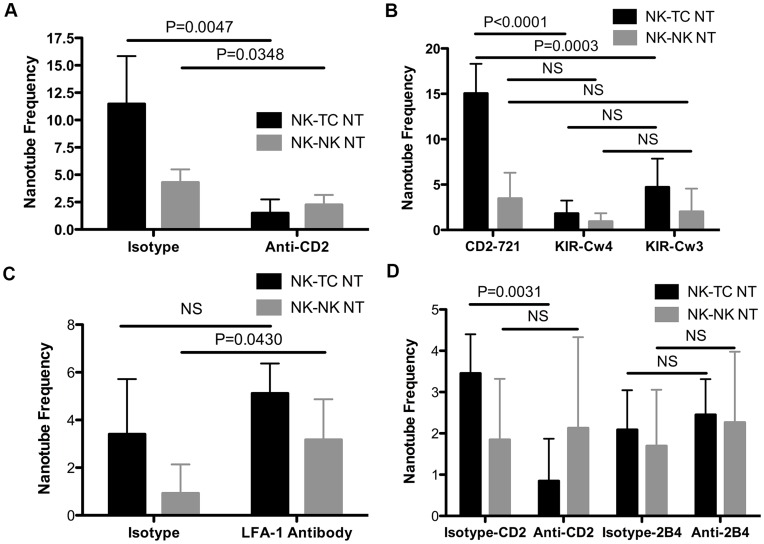
CD2, but not KIR2DL1, LFA-1, or 2B4, receptor-ligand interactions promote NT formation. (A) YTS-CD2-GFP cells were incubated with isotype or anti-CD2 blocking antibody for 15 minutes, followed by a 30 minute incubation with 721 target cells on anti-CD48 coated plates. Single time-point images were obtained to determine frequency of NT formation (n>350 cells). (B) YTS-KIR-GFP cells were incubated with 721-Cw4 cognate ligand-expressing cells or 721-Cw3 non-cognate ligand-expressing cells on anti-CD48 coated plates for 30 minutes. The frequency of YTS-CD2-GFP cells incubated with 721 cells (from Fig. 4A) is included for comparison (n≥300 cells). (C) YTS-CD2-GFP cells were incubated with isotype or anti-LFA-1 blocking antibody for 15 minutes, followed by a 30 minute incubation with 721 target cells on anti-CD48 coated plates. Single time-point images were obtained to determine frequency of NT formation (n>500 cells). (D) YTS-CD2-GFP cells were incubated with isotype, anti-CD2 blocking antibody, or anti-2B4 blocking antibody for 15 minutes, followed by a 30 minute incubation with 721 target cells on anti-CD20 and FN coated plates. Single time-point images were obtained to determine frequency of NT formation (n>500 cells). Bars show the percentage of NK cells exhibiting NK to target cell NTs (black) and NK to NK cell NTs (grey).

**Figure 6:**
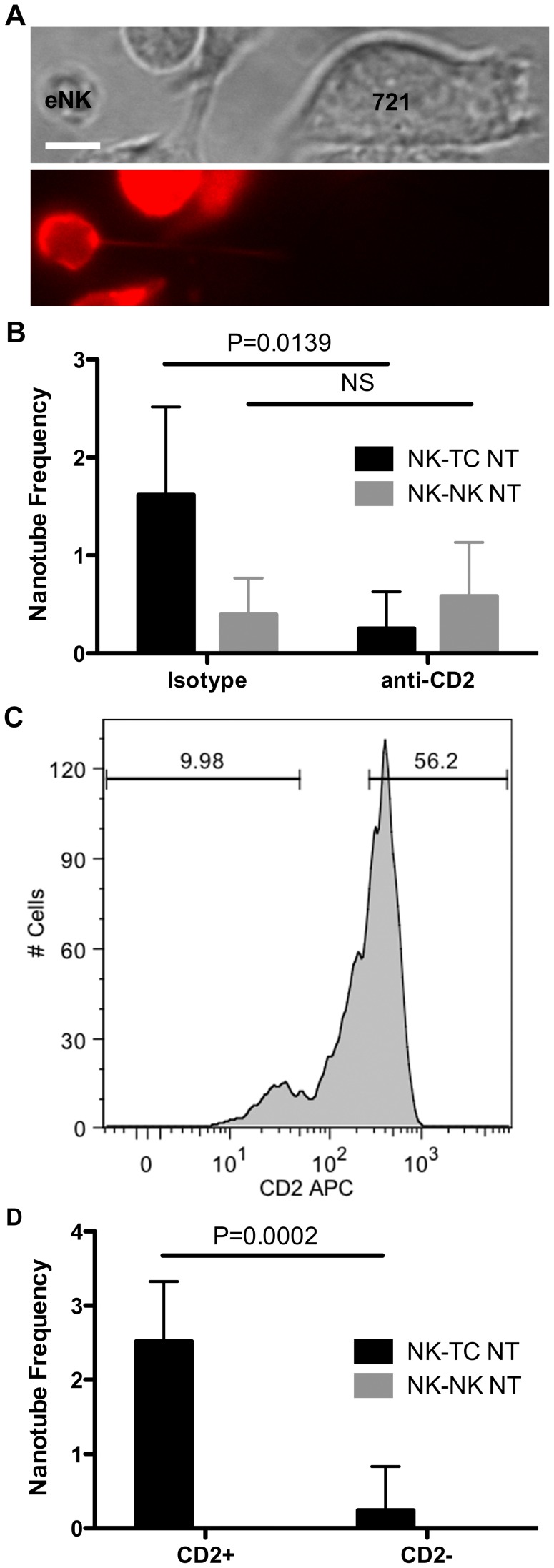
CD2 receptor-ligand interactions promote NT formation in eNK cells. (A) NTs were visualized in PKH26 labeled eNK cells isolated from whole blood. (B) eNK cells were labeled with PKH26, treated with isotype or anti-CD2 blocking antibody for 15 minutes, then incubated with 721 cells on anti-CD48 coated plates for 30 minutes. Single images were obtained to determine NT frequency (n>850 cells). (C) eNK cells were sorted into CD2^+^ and CD2^−^ populations using a non-blocking antibody to CD2. (D) CD2^+^ and CD2^−^ eNK populations were labeled with PKH26, and single images were obtained to determine the frequency of NK-NK (black bar) and NK-TC (grey bar) NT formation (n>325 cells). (Scale bar: 5 µm.).

The minimum number of cells to be evaluated in each microscopy experiment was determined using a sample size calculation based on preliminary data. Sample size calculations were performed using the DSS sample size calculator (DSS Research) and α and β errors of 1%. Statistical analysis was performed using Prism (GraphPad). All error bars represent the standard deviation. In Fig. 1D, individual ratios were analyzed using a Student’s two tailed *t* test while overall the data was analyzed using a paired two tailed *t* test. Fig. 2D was analyzed using one-way ANOVA as well as a series of individual *t* tests. Data in Figs. 2D, 3D, 3E, and 3F were fit using a linear regression model. The coefficient of determination was calculated for Fig. 2D, and Figs. 3D, 3E, and 3F were analyzed to determine whether the slope differed significantly from 0. Figs. 4A, 5A, 5B, 5C, 5D, 6B, and 6D were analyzed using two-way ANOVA followed by a series of individual *t* tests. Fig. 4B was analyzed using a Student’s two tailed *t* test.

## Results

### YTS-CD2-GFP but not YTS-MyoIIA-GFP NK Cells form Stable NTs

Primary human NK cells and the immortalized human NK cell line NKL readily form NTs between themselves as well as various target cells [16]. Our first attempts to observe NT formation employed YTS cells stably expressing a myosin IIA-GFP fusion protein (YTS-MyoIIA-GFP). The immortalized NK cell line YTS has a limited receptor-ligand repertoire and does not require the presence of cytokines for growth in vitro. We utilized myosin IIA-GFP expressing cells since myosin is an abundant cytoplasmic and cortical protein, which we hypothesized would provide an opportunity to observe the formation of NK to target cell NTs. While these cells adhere to and mediate cytotoxicity against 721 B-lymphoblastoid target cells [17], they did not form NTs observable by either DIC or fluorescence microscopy for myosinIIA-GFP (Fig. 1A). In contrast, others had shown that 6.3% of primary NK cells and 7.5% of NKL cells were connected to 721 cells by NTs [16].

In an attempt to better reproduce culture systems known to promote NT formation, imaging chambers were coated with fibronectin (FN) [16], which is implicated in NK cell motility [25], in addition to using the anti-CD48 to immobilize the 721 cells. We hypothesized that FN would facilitate NK cell motility and thereby increase cell-cell contacts. Both cell-cell contact [16] and a substratum supporting optimal cell motility [15] are considered central to NT formation. On FN coated imaging chambers, however, only 1.1% of all observed YTS-MyoIIA-GFP cells formed NTs. Furthermore, only 2.0% of all YTS-MyoIIA-GFP and 721 cell interactions (defined as physical contact between the two cells lasting longer than one minute) resulted in NT formation (Fig. 1A). NTs that did form lacked the stability and characteristics of NTs described elsewhere [10], [15], [16], [26]. The rare NTs observed typically broke free of the target cell within seconds of their detection, leaving a long membranous projection that was eventually reintegrated back into the NK cell (Fig. 1B). These rare NTs also exhibited unusually thick diameters (∼400 nm) as compared to those reported previously [10], [15], [16], [26].

Cytokine stimulation of NK cells has been shown to approximately double the frequency of NK cell NTs [16]. We hypothesized that by using an NK cell line that does not depend on exogenous cytokine for growth, we removed a signal necessary for NT formation. Since YTS cells respond to IL-2 [27], however, we tested this hypothesis, by stimulating YTS-MyoIIA-GFP cells with IL-2 for 24 hours prior to addition to target cells in anti-CD48 and FN coated imaging chambers. Surprisingly, this failed to increase the frequency of NT formation; in fact, no NTs were identified following IL-2 pre-activation (Fig. 1A).

Another distinguishing feature of YTS cells is that they lack the cell surface receptor CD2. Given that receptor-ligand interactions are important mediators of cell-cell adhesion and several interactions have been demonstrated relevant to NT formation [16], we hypothesized that CD2 is important in promoting NT formation. To test this, we utilized YTS cells stably expressing a CD2-GFP fusion protein. Interestingly, 9.8% of all YTS-CD2-GFP cells formed NTs and 14.3% of all YTS-CD2-GFP and 721 cell interactions resulted in NT formation. Unexpectedly, this percentage was reduced on FN coated image chambers, with only 3.9% of all YTS-CD2-GFP cells forming NTs and 5.0% of all YTS-CD2-GFP and 721 cell interactions resulting in NT formation (Fig 1A). The NTs observed in these experiments exhibited usual characteristics. They remained connected to target cells for long periods of time (sometimes over 2 hours) and had more typical diameters of ∼100 nm (Fig. 1C). This increase in NT formation suggested that the presence of CD2 in YTS cells promotes their ability to form NTs.

Consistent with the reported role for NTs in enhancing NK cell cytotoxicity by contributing to ∼17.4% of target cell deaths [16], NT-forming cells should exhibit similar enhanced killing compared with those unable to form stable NTs. We tested this hypothesis in aggregate using a ^51^Cr release assay to evaluate the cytotoxic activity of YTS-CD2-GFP and YTS cells. YTS cells exhibited less target cell lysis than YTS-CD2-GFP cells, with ∼16% less lysis at the largest effector to target cell ratio (Fig. 1D). While activation- and adhesion-promoting properties of CD2 are likely to contribute to this decrease in cytotoxic function, these results are also consistent with the previously reported role that NTs are described to serve in NK cell cytotoxicity [16]. In addition to being consistent with these previously published predictions, the present results support the observation that CD2-expressing NK cells stably form NTs.

### YTS-CD2-GFP Cells can Simultaneously Form Multiple NTs

Microscopy carried out after cells had been incubated together for 30 minutes revealed that a high percentage of YTS-CD2-GFP cells form more than one NT when interacting with target cells. Multiple NTs formed between an NK cell and a single target cell (Fig. 2A) or between an NK cell and multiple target cells (Fig. 2B). Approximately 40% of all NK cells with at least one NT formed more than one, and the number of cells exhibiting multiple NTs was inversely related to the number of NTs per cell (Fig. 2C). Importantly, multiple NTs were not identified between just two NK cells (n>320 cells), although a single NK cell could form NTs to multiple NK cells (data not shown). Thus the formation of multiple NTs between two cells was specific to interactions between NK and target cells.

The mean length of NTs emanating from a single NK cell was inversely related to NT number (Fig. 2D). For cells containing 1, 2, 3, or 4 NTs, the mean NT lengths were 12.5±7.3, 10.9±10.2, 8.4±5.8, and 3.2±1.3 µm, respectively, and could be described in aggregate by a linear trend line with a correlation coefficient of r^2^ = 0.93. This linear trend suggests there is a finite limit to how many NTs a single cell can maintain, supporting the hypothesis that NTs are created from a single membrane reservoir [28]. As the cell creates more NTs, it is presumably able to devote less membrane to each NT, ultimately resulting in shorter NTs. Furthermore, the shorter length of multiple NTs substantiates their previously proposed role in target cell traction or anchoring [16]. The observation that NK cells with 4 NTs were always close to the target cell also suggests that they can synergize in this capacity. Thus NK cells can likely fix to a susceptible target by taking advantage of multiple NTs.

While two-dimensional imaging allows for the detection of multiple NTs, they are likely to exist in orientations that are underestimated when viewed in a single plane. Thus we utilized three-dimensional imaging of living YTS-CD2-GFP cells to provide further detail. This approach demonstrated more NTs than can be identified in the plane of perceived maximal NT fluorscence in a two-dimensional image (Video S1). However, due to the limits of spatial resolution within a given plane, the number of NTs that escape identification using two-dimensional images is likely proportional to the number of NTs identified. In other words, more NTs will likely be missed in cells that contain a larger number of identifiable NTs. The method employed here of scanning through z-planes to identify NTs ensures that if an NK cell contains a NT it will be recognized, even if defining the exact number present is not feasible. This further supports our observations regarding NT frequency, as unexpected disproportional concentrations of NTs outside of the maximal two-dimensional fluorescence plane were not observed (n = 8 cell-to-cell interactions with NTs). Moreover the estimation of frequency only relies upon identification of NTs and not their total number.

### CD2 Localized to the Distal Tip of the NK Cell NT

Previous studies have shown that receptors associated with NT formation are found in increased numbers at the distal tip of the NT [16]. Similarly, we observed bright patches of fluorescence at the tips of YTS-CD2-GFP NTs, consistent with an enrichment of CD2 (Figs. 1C, 2A, 2B, 3A). To quantify this enrichment, we compared the mean fluorescence intensity of the NT tip to that of the cell body (Fig. 3B) and the NT as a whole (Fig. 3C). Approximately 65% of NTs exhibited an enrichment of CD2 at the tip compared to the cell body and 85% exhibited an enrichment compared to the overall NT. Enrichment persisted over the life of a NT and appeared to increase over time (Fig. 3A). While two-dimensional images provided enough detail to quantify this enrichment, a three-dimensional image demonstrates more clearly the magnitude of the CD2 enrichment at the NT tip (Video S1).

In order to accurately quantify time-dependent enrichment of CD2, we generated linear trend lines for CD2 fluorescence intensity. This was performed using time-lapse video sequences, which were corrected for photobleaching. Videos in which NTs exhibited substantive tip enrichment of CD2 were considered separately from those that did not exhibit enrichment (Fig. S1). We first examined sum fluorescent intensity at the NT tip in order to gain insight into the relative number of GFP molecules present over time (Figs. 3D and S1A). A majority (85%) of NTs exhibiting CD2 enrichment showed an increase in CD2 at the tip over time, as demonstrated by a positively sloped trend line for the sum fluorescent intensity. In contrast, all NTs that failed to exhibit CD2 enrichment showed a decrease in CD2 sum fluorescence intensity over time (data not shown). The decrease in this case was likely to have been a feature of time-dependent photobleaching. A time-weighted average of the slopes in which CD2 accumulated at the NT tip shows the average increase in sum fluorescent intensity to be 55847 a.u./min. Thus CD2 accumulated at and relocalized to the NT tip over time.

Since it appeared that increasing fluorescence of CD2 at the NT tip might not be limited to the original precise point of formation with the target cell, the video sequences used to define accumulation of CD2 were more closely examined. Quantitative evaluation of the area of CD2 fluorescence revealed that the tips of many NTs increased slightly in size over time (Fig. S1B). Most (77%) of NTs exhibiting CD2 enrichment showed a time-dependent increase in tip area, as demonstrated by the mild trend line slope (Fig. 3E). In contrast, all of the NTs that failed to exhibit CD2 enrichment showed a decrease in tip area over time (which, again, was likely to have been a feature of photobleaching, not shown). A time-weighted average of the slopes in which CD2 was enriched shows the average increase in tip area to be 0.0066 µm^2^/min. Finally, we examined the mean fluorescence intensity as a marker for CD2 density within a given area. Only 46% of NTs exhibiting CD2 enrichment showed an increase in CD2 density at the tip over time (Fig. S1C). This enrichment, however, was substantive enough to lead to a positively sloped trend line for the mean fluorescent intensity with the time-weighted average of these slopes showing the average mean fluorescent intensity to increase with a slope of 20630 a.u./(µm^2^)/min (Fig. 3F). 100% of the NTs that failed to exhibit CD2 enrichment showed a decrease in CD2 density (data not shown). Taken together, these data demonstrated that CD2 quantity and area at the NT tip is dynamic.

### YTS-CD2-GFP Cells Form More NTs than YTS-KIR-GFP Cells

Our initial attempts to observe NT formation in YTS-MyoIIA-GFP cells suggested that unmodified YTS cells may not stably form NTs and that CD2 may play a crucial role in NT formation. An alternative hypothesis is that fluorescently tagging CD2 (a membrane bound receptor) merely aided visualization of NTs. Since MyoIIA is not an integral membrane protein, perhaps YTS-MyoIIA-GFP NTs escaped detection. To ensure that NT formation was not simply a feature of exogenously expressing a GFP-fused membrane bound receptor, we compared the frequency of NT formation of YTS-CD2-GFP cells to that of YTS-KIR-GFP cells. While the 721 target cells utilized in both experiments contain the CD2 cognate ligands CD58 and CD48, they lack HLA-Cw4, the cognate ligand of KIR2DL1. As such, the KIR2DL1 receptor-ligand pair should not influence NT formation. Thus, both of these pairings represent an NK cell interacting with a susceptible target cell, and the YTS-KIR-GFP cells provide a control for an NK cell expressing a GFP-labeled surface receptor.

YTS cells and 721 cells were incubated for 30 minutes on an anti-CD48 coated plate and then imaged using live-cell confocal microscopy, and z-planes were scanned to ensure that all NTs were noted. 15.1±3.3% of YTS-CD2-GFP cells exhibited NTs, compared to just 2.5±1.5% of YTS-KIR-GFP cells exhibiting NTs (Fig. 4A). Importantly, no YTS-KIR-GFP cells formed multiple NTs, either with a single or multiple target cells. The low frequency of NT formation in YTS-KIR-GFP cells mirrored that of YTS-MyoIIA-GFP cells, confirming that NT formation between NK and susceptible target cells is not merely a function of exogenously expressed GFP-cell surface receptors. Interestingly, there was no statistical difference in the frequency of NK cell to NK cell NT formation between YTS-CD2-GFP cells and YTS-KIR-GFP cells (3.5±2.9% and 3.7±2.8%, respectively, Fig. 4A). This suggests that NK to NK cell NT formation may rely on different factors than does NK to susceptible target cell NT formation.

NT characteristics of YTS-CD2-GFP and YTS-KIR-GFP cells were also compared. While the number of NTs formed differed greatly between YTS-CD2-GFP and YTS-KIR-GFP cells, the mean NT length did not. YTS-CD2-GFP NTs had a mean length of 10.4±8.0 µm, while YTS-KIR-GFP NTs had a mean length of 9.3±6.5 µm (Fig. 4B). These values lacked statistical difference and agreed closely with the reported mean NT length of 11.4 µm for primary human NK cells [16]. In contrast, very few YTS-KIR-GFP NTs exhibited KIR enrichment at the distal NT tip (Fig. 4C and 4C inset), particularly compared to the strong enrichment seen with YTS-CD2-GFP cells (Fig. 4D). No YTS-KIR-GFP cells exhibited KIR tip enrichment compared to the cell body, and only 10% (1 NT) exhibited KIR enrichment at the tip compared to the overall length of the NT. These observations support the hypothesis that the CD2 receptor plays a specific role in NT formation. It also provides specificity regarding the observation that membranous CD2 preferentially relocates to the NT tip. Thus, it is likely that the interaction of CD2 with its ligand on target cells promotes NT formation.

### CD2, but not KIR2DL1, LFA-1, or 2B4, Receptor-ligand Interactions Promote NT Formation

To more directly address the role of CD2 in NT formation, we blocked CD2 receptor-ligand interactions using a blocking anti-CD2 mAb [20], [21]. YTS-CD2-GFP cells were incubated with mAb or isotype control for 15 minutes prior to incubation with 721 target cells on anti-CD48 coated plates. Again, thorough scanning through focal planes was performed to ensure NT detection. 11.5±4.4% of cells treated with isotype control Ab formed NTs with target cells as compared to only 1.5±1.2% of cells treated with the CD2 mAb (Fig. 5A). This decrease in frequency of NT formation to the level of YTS-MyoIIA-GFP and YTS-KIR-GFP cell NT formation supports the hypothesis that the specific interaction of CD2 with its ligand (which is highly expressed on 721 cells, not shown) plays a crucial role in NT formation. In contrast, 4.3±1.2% of isotype and 2.3±0.9% of mAb treated YTS-CD2-GFP cells formed NK to NK cell NTs (Figs. 5A). Although this difference is statistically significant, it represents a much smaller effect, suggesting that CD2-ligand binding may play a minor role in NK to NK cell NT formation.

NKG2D-MICA and LFA-1-ICAM receptor-ligand interactions have been described to participate in NT formation, suggesting that perhaps receptor-ligand interactions in general enable NT formation [16]. To investigate whether any form of receptor-ligand interaction can promote NT formation, we chose to examine the inhibitory KIR2DL1-HLA-Cw4 interation. YTS-KIR-GFP cells were added to 721 cells expressing either HLA-Cw4, or a homologous but non-cognate ligand, HLA-Cw3. Only 1.8±1.4% of YTS-KIR-GFP cells formed NK to target cell NTs with 721-Cw4 cells (Fig. 5B). This is remarkably lower than both the 15.1% frequency for YTS-CD2-GFP cells (Fig. 4A) and the 4.7±3.2% formed by YTS-KIR-GFP cells when mixed with 721-Cw3 target cells (Fig. 5B). Clearly not all receptor-ligand pairs promote the formation of NTs, with the inhibitory KIR2DL1-HLA-Cw4 interaction providing an example. The comparison of NT formation in YTS-KIR-GFP cells between 721-Cw4 and 721-Cw3 cells was not significant.

Because of the inhibitory nature of the KIR2DL1-HLA-Cw4 receptor-ligand interaction, it is not entirely surprising that it fails to promote NT formation. Thus we asked whether other activating and adhesion receptors also promote NT formation similarly to CD2. We initially targeted LFA-1 since, like CD2, it is well known to promote both adhesion and NK cell activation signaling [3], [4]. Previous work has demonstrated that enhancing LFA-1-ICAM interactions to supraphysiologic levels through the use of Mn^2+^ promotes NK cell NT formation [16], [29]. To determine if endogenous LFA-1 interactions play a role in NT formation, we abrogated LFA-1 receptor-ligand interactions using a blocking anti-LFA-1 mAb [22], [23]. Surprisingly, only 3.4±2.3% of cells treated with isotype control Ab formed NTs with target cells as compared to 5.1±1.2% of cells treated with the LFA-1 mAb (Fig. 5C). Thus, endogenous LFA-1 fails to play a role in NT formation in this context.

Next, we asked whether another CD2 family member, such as 2B4, may play a role in NT formation. 2B4, like CD2 and LFA-1, plays a role in NK cell adhesion [3], [4] and activation [2], [30]. Importantly, 2B4 shares CD48 as a cognate ligand with CD2, however in the case of 2B4, CD48 is its high-affinity ligand. Because of this, CD48 could no longer be utilized to immobilize the 721 cells to the imaging chambers. Instead, chambers were coated with anti-CD20 and FN. Because NK cells are more motile under these imaging conditions, they were more likely to form clumps of cells, and thus NTs were formed less frequently than on anti-CD48 coated imaging chambers. However, treatment with anti-CD2 blocking antibody still inhibited NT formation, with 3.5±1.0% of isotype control Ab treated cells and 0.8±1.0% of anti-CD2 mAb treated cells forming NK to target cell NTs (Fig. 5D). Additionally, treating YTS-CD2-GFP cells with a blocking anti-2B4 mAb [21] had no effect on NK to target cell NT formation, with 2.1±1.0% of isotype control Ab treated cells and 2.5±0.9% of anti-2B4 mAb treated cells forming NK to target cell NTs (Fig. 5D). This demonstrates that like LFA-1, endogenous levels of 2B4 fail to play a role in NK to target cell NT formation.

### CD2 Receptor-ligand Interactions Promote NT Formation in eNK Cells

Although the YTS cell line has faithfully reproduced the biology of human NK cells in a number of contexts (eg. [27], [31], [32]), we wanted to examine the effect of CD2 receptor-ligand interactions on NT formation in ex vivo NK (eNK) cells. eNK cells were labeled with the fluorescent membrane dye PKH26 and pretreated with either CD2 blocking mAb or isotype control for 15 minutes prior to incubation with 721 target cells. NTs were readily identified (Fig. 6A). 1.6±0.9% of eNK cells treated with isotype Ab formed NTs with target cells compared to only 0.3±0.4% of eNK cells treated with the CD2 blocking antibody (Fig. 6B). This decrease in NT frequency further supports the hypothesis that the CD2 receptor-ligand interaction promotes NT formation. 0.4±0.4% of eNK cells treated with isotype and 0.6±0.6% of those treated with CD2 blocking mAb exhibited NK to NK cell NTs. These latter results are not statistically different, supporting our previous hypothesis that CD2’s role, if any, in NK-NK NT formation is minor thus further substantiating a function for CD2 in promoting target cell-specific NTs in NK cells.

To further confirm the importance of CD2 in NK cell to target cell NT formation, we isolated highly purified CD2^+^ and CD2^−^ eNK cell populations by FACS using a non-blocking CD2 antibody (Fig. 6C) [20]. To confirm that this antibody did not interfere with NT formation, it was first evaluated for any effect on the formation of NTs when added to YTS-CD2-GFP cells combined with susceptible 721 target cells. While YTS-CD2-GFP cells treated with the blocking antibody demonstrated an 88.0±4.7% decrease in NT frequency to target cells compared to cells treated with an isotype control, treatment with the non-blocking anti-CD2 antibody resulted in only a 5.0±13.0% decrease (p = 0.001, n>325 cells). Therefore, eNK cells sorted using this non-blocking anti-CD2 antibody were used to determine if the presence of CD2 might enable NT formation in fresh human NK cells. Directly following sorting using the non-blocking antibody, the CD2^+^ and CD2^−^ eNK cells were labeled with PKH26 and incubated with 721 target cells for 30 minutes. 2.5±0.8% of CD2^+^ eNK cells formed NK to target cell NTs, while 0.2±0.6% of CD2^−^ eNK cells formed NTs (Fig. 6D). No NK to NK cell NTs were observed. Taken together, the above experiments demonstrate that the presence of CD2 and the CD2 receptor-ligand interaction play an essential role in enabling NT formation in NK cells directly ex vivo in their interaction with a susceptible target cell.

## Discussion

These are the first data to demonstrate NT formation in YTS NK cells, but more importantly have thereby identified a receptor that plays a crucial role in NT formation. Previous research has documented NT formation in immortalized NKL cells, however those NTs differ from the ones formed by primary NK cells. NKL cell NTs have a much longer average length (21.4 µm) than do primary NK cell NTs (11.4 µm) [16]. The average length reported here in YTS cells (∼10 µm) is more consistent with that of primary NK cell NTs, suggesting that YTS cells may form NTs more structurally similar to those of ex vivo NK cells.

Because the YTS cell line inherently lacks CD2, it provided a fortuitous way to observe the effects of the CD2 receptor-ligand interaction on NT formation. YTS-CD2-GFP cells formed more NK to target cell NTs than did either YTS-MyoIIA-GFP or YTS-KIR-GFP cells, and blocking CD2’s interaction with its ligand through the use of a blocking antibody abrogated NK to target cell NT formation. Interestingly, the use of a non-blocking antibody had no impact on NT formation, suggesting that the CD2-CD58/48 receptor-ligand interaction plays a crucial role in NT formation. CD2 expression also increased the cytotoxic activity of YTS cells at levels commensurate with what has been previously attributed to the cytotoxicity promoting capacity of NTs [16]. Taken together, these data suggest that CD2 plays an essential role in the formation of NTs, which are known to enhance NK cell cytotoxicity [16].

We also show that CD2 plays a role in NT formation ex vivo, as blocking CD2 on eNK cells dramatically reduces the frequency of NT formation between NK cells and susceptible target cells. In addition, CD2^−^ eNK cells form NTs with targets at a negligible frequency whereas CD2^+^ NK cells have robust numbers of NTs. Therefore, we show for the first time that CD2 plays a crucial role in the formation of NTs both in a cell line and ex vivo human NK cells.

Using YTS-CD2-GFP cells, we demonstrated that, like NKG2D [16], CD2 is enriched at the NT tip. Through analysis of time-lapse video sequences, we also provided evidence that this enrichment occurs over time. The accumulation of CD2 at the NT tip suggests that it may play a role in maintaining NT stability. Alternatively, CD2 signaling may drive NT function, facilitating NT-mediated reformation of the conventional NK cell immunological synapse or activating long-range cytotoxic activity, both of which have been previously proposed as possible NT functions [16].

Interestingly, we found that only interactions between NK cells and target cells result in the formation of multiple NTs and that the number inversely correlates with their length. In other words, more NTs result in a shorter distance between the NK cell and its target. When NK cells possessing 4 NTs with a single target cell were identified, the intercellular distance was uniformly small. These findings further support the proposed function of NK cell NTs in facilitating contact with target cells. Additionally, the presence of CD2 was needed for multiple NT formation with a target cell as the few MyoIIA-GFP and KIR2DL1-expressing cells that formed NTs with target cells failed to demonstrate multiple intercellular NTs. Thus the presence of multiple NTs between an NK cell and target is suggested as a correlate of the previously proposed NT enhanced cytotoxicity [16] since the YTS-CD2-GFP cells had increased cytotoxic activity. At a minimum, multiple NTs between an NK cell and target cell can be viewed as a hallmark of a potentially cytotoxic interaction.

We also demonstrate that not all receptor-ligand interactions promote NT formation, with KIR2DL1-HLA-Cw4 providing an example. Since this receptor-ligand pair provides an inhibitory signal, it will be important to explore if there might indeed be a decrease in NT frequency measured in the presence of the KIR2DL1-HLA-Cw4 ligation. Although the difference in NT formation in KIR2DL1-expressing YTS cells between targets expressing the cognate or non-cognate ligand was not significant, it raises the possibility that inhibitory signaling could interfere with NT formation. The converse logic suggests that, aside from receptor-ligand interaction, an activation signal is actually needed for NT generation, and certainly for the generation of multiple NTs between NK and target cells.

Taken together, these results suggest that CD2 plays a crucial role in NK cell NT formation. Indeed, we also demonstrate that the activating and adhesion receptors LFA-1 and 2B4 fail to play as critical a role, since blocking these receptor-ligand interactions does not decrease NT formation. In fact, blockade of both receptors resulted in a potentially small, although statistically insignificant, increase in NT formation. Most likely, this is the result of decreased adhesion interactions increasing NK and target cell separation events, and thus increasing the formation of NTs. Surprisingly, our results of blockading LFA-1 interactions contrast previous reports that enhancing LFA-1 interactions through the use of Mn^2+^ promotes NK cell NT formation [16]. Our results, however, are limited to otherwise unactivated LFA-1 without an increase in LFA-1-ICAM interactions owing to the use of Mn^2+^. It is possible that in highly activated cells the role of LFA-1 is distinct. Thus, our data suggests that only specific receptor-ligand interactions, including but not limited to NKG2D [16] and CD2, promote NK to target cell NT formation. Further research is needed to fully appreciate why these specific receptors play such an essential role, their relative context-specific contributions, and if any other receptors provide similar promotion of NT formation.

Our results are inconclusive on whether CD2 influences NK to NK cell NT formation. While YTS-CD2-GFP cells treated with a CD2 blocking antibody formed slightly fewer NK to NK cell NTs than did isotype control treated cells, eNK cells treated with CD2 blocking antibody formed the same number of NK to NK cell NTs as isotype control treated eNKs. Similarly, there was no significant difference in NK to NK cell NT formation between YTS-CD2-GFP and YTS-KIR-GFP cells. This difference between NK to NK cell and NK to target cell NT formation is intriguing, particularly since NK cells express both CD48 and CD58 [21]. Future research is needed to better understand this difference and the molecular interactions governing NK to NK cell NT formation.

T cells [15], [26], B cells [33], [34], dendritic cells [14], macrophages [35], neutrophils [36], and NK cells all form NTs. Some or all of these cells may use NTs to communicate and coordinate immune responses. Through NT coordination, B cell activation could be transmitted to distant T cells [33], activating signals could be generally transmitted to a whole network of NT-connected cells [14], or, in the case of NK cells, NK-dendritic cell crosstalk and the transmission of cytokines, both to and from the NK cell, could be mediated over long distances via NTs. Interestingly, CD2’s cognate ligand, CD58, is widely expressed on hematopoietic cells, making it an ideal candidate for promoting NT formation and subsequent crosstalk between a variety of immune cells. While the role for CD2 in NT formation in other immune cells remains unexplored, we demonstrate for the first time that the CD2 receptor-ligand interaction plays a crucial role in NK to target cell NT formation. In conjunction with the previously reported role for NTs in facilitating a fraction of NK cell cytotoxic function [16], our observations support the longstanding observation that CD2 serves as a costimulator for NK cell function [2] and implies a special utility of this receptor in enabling NK cell exploration of local environments through promoting NTs.

## Supporting Information

Figure S1 **Fluorescent intensity and area graphs for individual NTs observed over time.** (A) The sum fluorescent intensity, (B) area and (C) mean fluorescent intensity for each of the NTs used in calculating the graphs for Figs. 3D, 3E, and 3F are shown as a feature of observation time. Corresponding NTs are shown in the same color across the three graphs. NTs failing to exhibit tip to NT length CD2 enrichment (n = 2) are shown in red.
(TIFF)

Video S1 **Three-dimensional NT image demonstrates magnitude of CD2 tip accumulation and presence of multiple NTs.** A three-dimensional image of YTS-CD2-GFP cells conjugated with 721 cells for 30 minutes on anti-CD48 coated plates. Initial image shows a single z-plane with the 721 cell outlined in white and all cells labeled with text, followed by the three-dimensional image. The transition from the two, to three-dimensional sequence is noted by a “fade-out” effect. The three-dimensional image highlights that two-dimensional imaging fails to recognize the total number of NTs. It also highlights the large accumulation of CD2 at the tip of the long NT (scale bar = 9 µm).
(MOV)
