# Supplementary figures and images for: CD2 Promotes Human Natural Killer Cell Membrane Nanotube Formation
Source: PLoS One. 2012 Oct 24;7(10):e47664. doi: 10.1371/journal.pone.0047664 (PMC3480409; doi:10.1371/journal.pone.0047664)

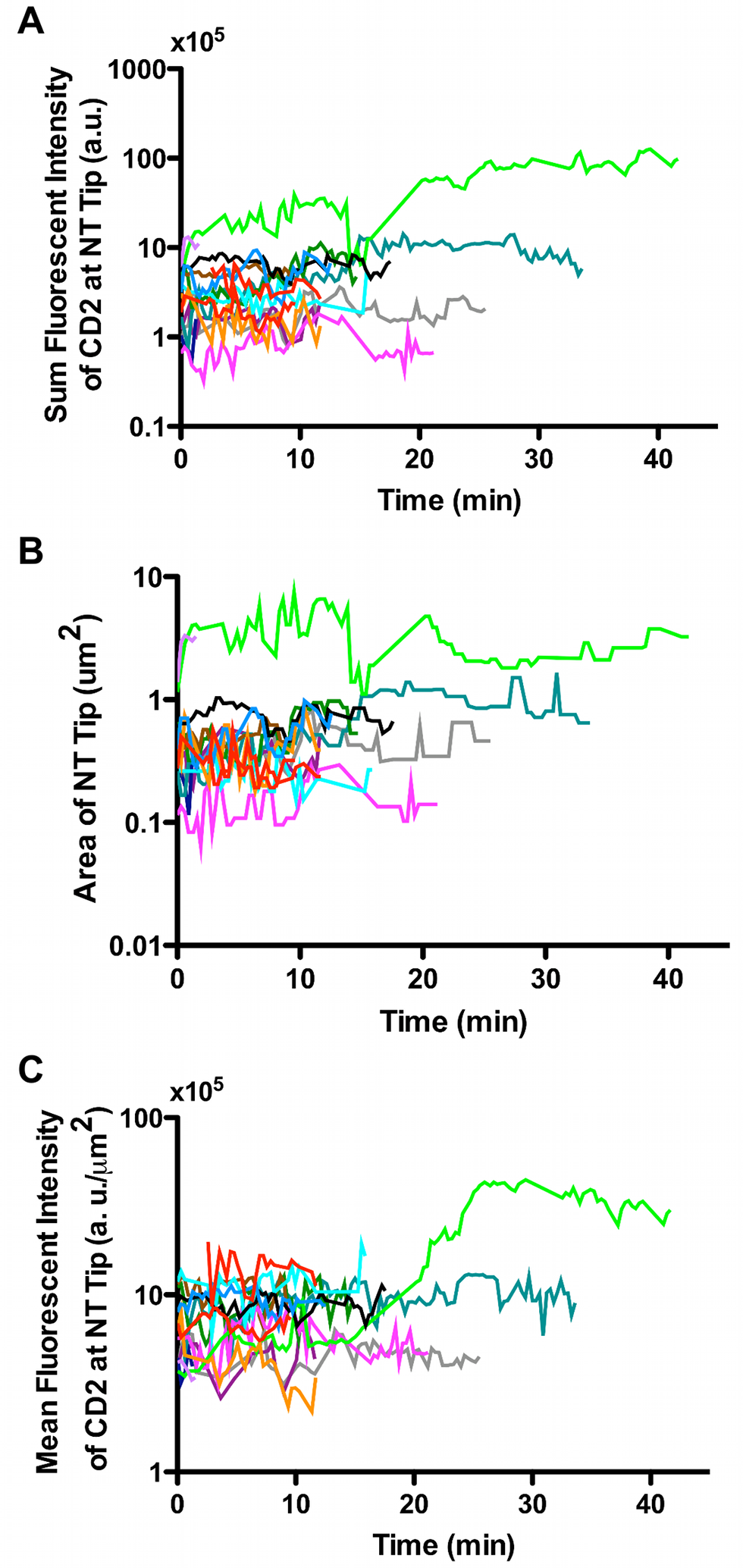

Supplement: Figure S1 — Fluorescent intensity and area graphs for individual NTs observed over time. (A) The sum fluorescent intensity, (B) area and (C) mean fluorescent intensity for each of the NTs used in calculating the graphs for Figs. 3D, 3E, and 3F are shown as a feature of observation time. Corresponding NTs are shown in the same color across the three graphs. NTs failing to exhibit tip to NT length CD2 enrichment (n = 2) are shown in red. (TIFF) [file pone.0047664.s001.tiff]
